# Flexible working in the UK and its impact on couples’ time coordination

**DOI:** 10.1007/s11150-017-9389-6

**Published:** 2017-09-27

**Authors:** Mark L. Bryan, Almudena Sevilla

**Affiliations:** 1 Department of Economics, University of Sheffield, 9 Mappin Street, Sheffield, S1 4DT UK; 2 Almudena Sevilla, Professor of Economics, School of Business and Management, Queen Mary University of London, Francis Bancroft Building, Room 4.13D, Mile End Road, London, E1 4NS UK

**Keywords:** Flexible work, Time synchronization, Time coordination, Work synchronization, Work coordination, Labor supply, J12, J22, J32

## Abstract

The ability to combine work with quality time together as a family is at the heart of the concept of work-life balance. Using previously unexploited data on couples’ work schedules we investigate the effect of flexible working on couples’ coordination of their daily work schedules in the UK. We consider three distinct dimensions of flexible working: flexibility of daily start and finish times (flexitime), flexibility of work times over the year (annualized hours), and generalized control of working hours. We show that having flexitime at work increases a couple’s amount of coordination of their daily work schedules by a half to 1 h, which is double the margin of adjustment enjoyed by couples with no flexitime. The impact is driven by couples with children. In contrast to flexitime, the other two forms of flexible working do not seem to increase synchronous time. Our results suggest that having flexitime plays an important role in relaxing the work scheduling constraints faced by families with young children, and that effective flexible working time arrangements are those that increase the worker’s and not the employer’s flexibility.

## Introduction

The choice and convenience in hours of work is at the heart of work-life balance, which goes beyond the total time available for out-of-work activities and crucially rests on being able to coordinate time with others, for example in order to enjoy more leisure time together. Previous literature has suggested that spouses’ work hours coincide more than they would if individuals were randomly paired up, suggesting that spouses have preferences for time together and do indeed actively coordinate their work schedules. However, these studies have not analyzed the mechanisms by which couples achieve coordination. In this paper we focus on one possible mechanism, flexible working.

Although worker-oriented flexible work arrangements (such as flexitime) may be able to facilitate coordinating time with others outside work by providing more control over working times, in the UK the prevalence of flexible work remains uneven across industries and types of workplace (Tipping et al. 2012). Under half of employees report access to flexitime (Nadeem and Metcalf 2007; Tipping et al. 2012), and lack of flexible work options have resulted in widespread pent-up demand for flexible working (Golden 2015). The UK ranks relatively low with respect to other countries in terms of work–life balance (see Bloom and Van Reenen 2006 study using an international dataset on work–life balance, which includes the firm’s provision of flexible work arrangements).

How much more synchronization there might be if individuals had more control over their work hours and the timing of work is not well understood. This paper uses previously unexploited data on time scheduling in the household and employment contexts to investigate for the first time the effect of flexible working on couples’ coordination of their work schedules in the UK.

We augment the standard neoclassical household coordination model by introducing flexible work as a fine-tuning mechanism that allows workers to choose a more efficient synchronous family time. In the model, couples choose from a set of discrete working schedules, which together imply a level of synchronous time. For example, if one spouse works 9am–5pm and the other works 10am–6pm, synchronous working time is 7 h (from 10am to 5 pm). But if one of the spouses has flexitime (variable start and finish times) they can adjust their working hours to be nearer their partner’s. If working hours coincide perfectly (both work 9–5) then synchronous time rises to 8 h. All else equal (including total working hours) the more synchronous work time couples have, the more time they can potentially spend together. We thus hypothesize that couples with flexible work arrangements should have more synchronous time than couples without.

A key factor in the calculation is the presence of children: as we explain, children introduce practical difficulties in coordinating schedules because of childcare requirements and other constraints. Thus, parents are able to coordinate their work schedules less than they would ideally like to. Previous studies indicate that couples with children get significantly less synchronous times than childless couples, and we find the same in our data. But it is not known how flexible work interacts with the presence of children. There is evidence that even couples with children coordinate to have more synchronous time than they would otherwise get.^1^ Thus if couples with children are further away from their optimal amount of synchronous time (e.g., because of childcare constraints), we hypothesize that flexible work should have a larger impact than on childless couples.

We test these predictions using the 2013 wave of the British Household Panel Survey (BHPS), which contains rich information about individuals and the households they belong to, including details of individuals’ labor market experiences and use of flexible work (as measured by several indicators in the data). In this wave the survey also contained the times respondents usually start and finish work, which we use to construct the length of time per day that partners coincide at work as a measure of how they synchronize their work schedules.

Our results from estimating an equation of couples’ coordination of their work schedules augmented with flexibility suggest that flexitime is associated with a half to 1 h increase in daily synchronous work time. This is a large figure given previous findings showing that the overall amount of synchronization is about an hour per day, irrespective of whether couples have flexitime or not (Hallberg 2003). In contrast to flexitime, neither a broader measure of work hours control nor the provision of annualized hours is strongly associated with a couple’s work schedule coordination. These findings could be explained by the fact that these arrangements may involve flexibility over a different timescale to flexitime, and thus any impacts from these alternative measures of job flexibility may be reflected less strongly in our measure of daily synchronous work time. An alternative interpretation could also be that arrangements such as annualized hours capture firm-oriented flexibility, which is less conducive to achieving work-life balance, rather than worker-oriented flexibility (Messenger 2011; Kerkhofs et al. 2008). Our results are robust to controlling for a wide set of household socio-economic characteristics, such as partners education and age, as well as total working time.

Consistent with our theoretical framework, we find that the positive effect of flexitime on couple’s synchronization is driven by couples with young children, partly via choice of occupations with greater flexitime. Families with young children may face a variety of additional constraints in the labor market with respect to couples without children because of the limited provision of childcare (Fermanian and Lagarde 1999), the timing-sensitive nature of children’s caring needs (Barnet-Verzat et al. 2011), and the higher costs associated with geographical mobility (Rabe 2011). Thus they face a smaller choice of jobs that may suit their work hours requirements. Using an IV strategy, we also find a causal impact of having a job with flexitime on couple’s synchronization, particularly for couples with younger children.

This paper extends the economics literature in three important ways. First, it adds to the emerging literature in labor economics on family friendly work practices, which include the provision of flexible working hours and annualized hours analyzed here. A growing body of research has investigated the impact of working time arrangements on outcomes such as firm performance, labor productivity and labor turnover (Dex et al. 2001), job satisfaction and organizational commitment (Martinez-Perez 2016; Scandura and Lankau 1997), and work-life balance (Hill et al. 2001). Rather than taking the perspective of firms or individual workers, here we take a household perspective to see how flexible working may affect couples’ work scheduling decisions.

Second, we add to the international literature documenting greater synchronization of work schedules between partners than would be expected from random pairings (see Hamermesh 2002 for the US; Jenkins and Osberg 2005 for an earlier study on Britain, van Klaveren and van den Brink 2007 for the Netherlands; and Scheffel 2010 for Germany). We first show that flexible work is an important determinant of the ability of couples to synchronize work schedules, and second we look at the importance of family structure in mediating the role of flexible work. Hallberg (2003) uses time-use data from Sweden to confirm that spouses specifically synchronize leisure time.

Third, we incorporate restrictions on workers’ ability to choose their work schedules into a standard neoclassical labor supply model and look at an additional dimension of time beyond the total time devoted to market work, i.e., the timing of market work, into the decisions taking place within the household. A limited set of studies have modeled the individual choice to work or not at different times of the day (see for example Hamermesh 1999). This paper expands this growing literature by incorporating a household dimension to the timing of work in order to study the coordination of work schedules between spouses, and by providing a theoretical link to the availability of flexible work.

The paper is organized as follows. Section 2 presents the theoretical framework on which the empirical analysis is based. Section 3 presents the British Household Panel Survey (BHPS) data and gives an overview of the distribution of schedule coordination and flexible work. Section 4 introduces the empirical specification and Section 5 presents the main results, while Section 6 concludes.

## Theoretical framework

Models of household work coordination (e.g., Hamermesh 2002; Scheffel 2010) extend conventional labor supply models by distinguishing between the utility that each partner in a couple derives from leisure (or non-work) time alone and the utility they derive from joint leisure (non-work time). Couples choose their work times to maximize overall utility and the resulting amount of synchronous work time (*h* ^*^) depends on the partners’ wage rates (*w* ^*m*^_,_ *w* ^*f*^) as well as personal and household characteristics (*X*):^2^

1 $${h^*} = {h^*}\left( {{w^m},{w^f};\,X} \right)$$

The elements of *X* capture the couple’s tastes for spending time together as well as other factors that may affect synchronous time. In particular, to the extent that couples aim to produce higher quality children, then having children may reduce synchronous time if, for example, the rigidities in childcare provision mean that one parent always has to stay with the child.

An implicit assumption in these models is that workers can choose from a wide range of different work schedules, either within the job or by moving (costlessly) between jobs. Observed work timings are then taken to reveal couples’ preferences for coordination given their earnings and household structure. Indeed, in these models there is little reason to investigate the impact of flexible work because all workers are already fully flexible.

To consider the likely impact of flexible work it is useful to think of a labor market with some constraints on hours that arise because employers have preferences over working time (perhaps because of the need to coordinate hours within work teams, or if productivity varies with working time as in Barzel 1973) and there are search or mobility frictions that prevent workers from costlessly changing jobs. Instead of a free choice of hours, we assume that workers are faced with a finite set of fixed hours schedules (Altonji and Paxson 1986; Dickens and Lundberg 1993). There is indeed evidence to suggest that there are significant constraints in the ability of workers to choose their desired working hours and degree of flexibility (Bryan 2007; Golden 2015; Böheim and Taylor 2004; Nadeem and Metcalf 2007).

The set of available schedules implies a discrete set of possible synchronous work times, for example *H* = {*h*_1_, *h*_2_, *h*_3_}. A couple will choose *h*∈*H* to yield the highest utility, however in general *h* will differ from their optimal synchronous time *h* ^*^. In this context flexible work can act as a fine-tuning mechanism that allows *h* to be brought closer to *h* ^*^. Thus the difference between the optimal synchronous time *h* ^*^ and the chosen amount of synchronous time *h* will generally be a function of the degree of flexibility in the market. Rather than Eq. (1), observed synchronous time is described by:

2 $$h = g\left( {{h^*},\,flex} \right) = \overline h \left( {{w^m},{w^f},X,flex} \right)$$

where *flex* indicates the degree of flexibility available to couple. Since the evidence from randomly-matched couples suggests a preference for more time coordination, we hypothesize that flexible work should be associated with more synchronous time.

In our expanded model, having children potentially affects time coordination by a second route. Complications around childcare timing may reduce range of jobs that parents can do and hence their choice set of schedules, such that for example *H* = {*h*_1_, *h*_3_} rather than {*h*_1_, *h*_2_, *h*_3_}. If this means that couples with children are further away from their optimal amount of synchronous time, we hypothesize that flexible work should have a larger impact than on childless couples.

In Section 5 we estimate a linearized version of (2) and test all of these hypotheses.

## Flexible working and couple’s work schedules in the UK

We use wave 13 from the British Household Panel Survey (BHPS). The BHPS is a longitudinal household survey that began in 1991 with a random sample of about 5000 private households, with additional samples of 1500 households in each of Scotland and Wales added in 1999, and a sample of 2000 households in Northern Ireland added in 2001.^3^ Wave 13 additionally contains information on work schedules for each member of the household. We look at couple households in which both (married or cohabiting) partners work full time (FT) or in which the male partner works full time and the female part time (PT) (we do not analyze couples containing a part time man, since these make up only around 2% of working couples, or same-sex couples).^4^ We have 1533 couples with valid shift times (778 without dependent children and 755 with children).

The analysis focuses on the extent to which partners synchronize their working schedules as a result of job flexibility. To that end we construct a measure of couples’ synchronous work time, which is derived from a special module to investigate work timing in wave 13 of the BHPS (2003). In this wave employees were first asked whether they worked the same hours each day, rotating shifts, or no fixed pattern. Respondents working the same hours each day (*regular workers*) were asked for the times they usually started and finished work, while those on rotating shifts or with no fixed pattern (*irregular workers*) were asked for start and finish times on each day of the preceding week (all times reported to the nearest minute). Using the reported times, we calculate our measure of synchronous working time as the amount of time per day during which both spouses work simultaneously. For example, if one spouse works 9am–5pm and the other works 10am–6pm, synchronous working time is 7 h (from 10am to 5 pm). The full wording for all questions and details of the calculation of synchronous time are in Appendix 1, Table 6. Similar to our study, Hamermesh (2000) and van Klaveren and van den Brink (2007) use survey questions on work start and finish times. Hallberg (2003) and Scheffel (2010) use time diary data, whose main advantage in this context is to distinguish between different non-work activities. Like us, Jenkins and Osberg (2005) used the BHPS but they used a binary measure of synchronization based on discrete indicators of work schedule (morning, afternoon etc); continuous measures of work time were not available in their data (1991–1999).

**Fig. 1:**
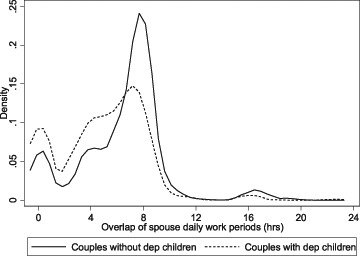
Overlap of work schedules within couples

The resulting measure of work overlap is a quasi-continuous variable and is shown in Fig. 1 as a kernel density plot, distinguishing between couples with and without dependent children.^5^ The amount of work overlap is less than 10 h for almost all couples (the overall mean is 5.7 h) with a notable spike at about 8 h for couples without children (mainly consisting of spouses who both work standard FT hours) and a less pronounced spike at about 7 h for couples with children. There are much smaller spikes for both groups at 0 h, corresponding to spouses who are never at work simultaneously. Unsurprisingly this arrangement is more common among couples with children (probably, as discussed later, reflecting ‘tag-team’ arrangements to ensure that one parent is always available for childcare). Apart from these spikes in the data there appears to be wide variation in work overlap times across couples (the overall standard deviation is 3.3 h).

We construct three measures of flexible working, each corresponding to a different type or dimension of flexibility. Two of the measures are derived from a BHPS question asking respondents to report, from a show card list, which working hours arrangements they have (see the full question wording in Appendix Table 7). The first measure is flexitime, which means choosing daily work times subject to being present during certain core hours each day (e.g., 10am–4pm).^6^ The second measure is annualized hours, meaning that employees must work a specified number of hours over the year but have some flexibility about when they work (possibly also subject to the level of demand by their employer). For the third measure we use a separate question asking respondents whether their work hours were decided by the employer, the respondent or both jointly. We define respondents who have at least some flexibility over working hours, or decide them jointly with their employer, or decide their hours themselves, as having control over working hours.

Panel A in Table 1 summarizes the distribution of these flexible practices for all couples, and the panels B and C for couples without and with dependent children. Overall, the table shows there is a high degree of heterogeneity in flexible working, both across couples and between partners within the same couple—such variation is crucial to establish whether it matters which partner has access to flexible work. In 14% of all couples, (only) the woman works flexitime, in 11% (only) the man works flexitime, and in a further 5% of couples both partners work flexitime. Thus, 30% of couples are able to use flexitime to some extent, with the female partner having more access to flexitime than her spouse. Annualized hours are less common, affecting only 14% of couples, with men slightly more likely to use them (men work annualized hours in 9% of couples compared with women in 7% of couples). By contrast, in the majority of couples men and women report they have at least some control in setting their hours (55% of couples for men and 58% for women), and in 35% of the couples both partners report they have this flexibility. Panels B and C show that there are relatively minor differences in the flexibility measures between couples with and without dependent children. The main exception relates to working hours control. In 33% of couples without dependent children both partners have at least some control over their working hours, compared with 38% of couples with children. Further investigation (not reported) indicates that this difference is largely driven by higher levels of hours control among part-time workers, who are more likely to have children (indeed alternative tabulations of flexible working broken down by dual FT vs. FT-PT status are very similar to those in Table 1).

**Table 1:** Flexible working practices within couples (%)

Within-couple prevalence of flexible working	Flexitime	Annualized hours	Working hours control
Panel A: all couples (*N* = 1533)			
Neither partner	70.5	86.5	22.6
Woman only	14.2	4.3	22.8
Man only	10.7	6.6	19.4
Both partners	4.6	2.6	35.2
Panel B: couples without dependent child(ren) (*N* = 778)			
Neither partner	72.2	86.3	25.8
Woman only	13.6	4.3	20.8
Man only	10.7	5.8	20.6
Both partners	3.5	3.6	32.7
Panel C: couples with dependent child(ren) (*N* = 755)			
Neither partner	68.7	86.6	19.4
Woman only	14.9	4.3	24.7
Man only	10.8	7.5	18.2
Both partners	5.7	1.6	37.7

Estimates weighted to account for survey design and non-response

Table 2 shows how couples’ synchronous working varies by the different flexible working practices. The asterisks indicate estimates, which are significantly different (at the 5% level) compared to couples without any flexible working. We see a positive and quite strong association between flexitime and synchronous work time, in particular for couples with children. For couples as a whole (panel A), couples in which neither partner has flexitime have 5.4 h of synchronous working time, increasing to 6.4 h if (only) the woman has flexitime, 6.1 h if (only) the man works flexitime, and 6.0 h if both have flexitime. All differences except the last are statistically significant. We find similar patterns for flexitime among couples with and without children but the associations are only significant for couples with children. Panel C shows that these couples work synchronously for 4.7 h when neither partner has flexitime, 5.8 h when the woman has flexitime, 5.4 h when the man works flexitime, and 5.8 h if both partners do (all differences with respect to couples without flexitime are significant).

**Table 2:** Couple’s synchronous working time (hours per day) by flexible working practices

Within-couple prevalence of flexible working	Flexitime	Annualized hours	Working hours control
Panel A: all couples (*N* = 1533)			
Neither partner	5.44	5.69	5.34
Woman only	6.44*	5.43	5.28
Man only	6.06*	5.54	5.41
Both partners	5.99	5.69	6.28*
Panel B: couples without dependent child(ren) (*N* = 778)			
Neither partner	6.39	6.46	6.35
Woman only	6.78	7.11	6.19
Man only	6.55	6.53	6.22
Both partners	7.10	6.38	7.03*
Panel C: couples with dependent child(ren) (*N* = 755)			
Neither partner	4.67	5.08	4.77
Woman only	5.76*	4.06*	4.59
Man only	5.44*	4.20	4.59
Both partners	5.83*	5.70	5.67*

Estimates weighted to account for survey design and non-response

* Denotes estimate is significantly different at 5% level from estimate for couples where neither partner works flexibly (*t*-test)

Compared to flexitime, the relationships appear less consistent for the other two flexible work measures. For annualized hours, the differences in synchronous time vary in sign and are only significant in one case (couples in which the woman works annualized hours synchronize less). Working hours control is typically associated with less synchronous time when it is available to only one partner (but these differences are not significant) and more synchronous time when both partners have some control over working time (this relationship holds whether or not couples have children).

## Flexible working and couple synchronization of work schedules

We use a multivariate analysis based on a linearized version of the synchronous time Eq. (2). The outcome is measured at the couple level and modeled as a function of both spouses’ characteristics and other household factors:

3 $${y_c} = {\beta _0} + {x_{1c}}\prime {\beta _1} + {x_{2c}}\prime {\beta _2} + {x_{3c}}\prime {\beta _3} + {f_{1c}}\prime {\gamma _1} + {f_{2c}}\prime {\gamma _2} + {\varepsilon _c}$$

where *y*_*c*_ is a measure of the amount of synchronous working time (in hours per day) experienced by couple *c*; *x*_1*c*_ and *x*_2*c*_ are vectors of characteristics associated with each spouse respectively and *x*_3_ contains household characteristics; *f*_1*c*_ and *f*_2*c*_ are measures of flexible working by each spouse; and *ε*_*c*_ is a random error term. In line with previous studies of synchronous time use (Hamermesh 2002; Hallberg 2003; Jenkins and Osberg 2005), vectors *x*_1*c*_ and *x*_2*c*_ contain explanatory variables for desired synchronous time such as spouses’ age, education and wages, and *x*_3c_ includes the presence and ages of dependent children. Geographical controls capture the effect of other cofounders, such as regional unemployment levels (see Morill and Pabilonia 2015), which may be correlated to both flexibility at work and couple’s synchronization. In extension specifications we also include occupation, industry and public/private sector dummies to control for demand-side constraints and also explore mechanisms of selection into flexible work. The variables used in the analysis are summarized in Appendix Table 8.^7^ Given the quasi-continuous nature of the synchronous time measure, we estimate (3) by OLS.

The key parameters to be estimated are *γ*_1_ and *γ*_2_, which show how flexible working by each spouse affects the amount of synchronous time enjoyed by the couple.^8^ Comparing the relative magnitudes of *γ*_1_ and *γ*_2_, we can test for differences across partners in the effects of flexible working on the couple’s time together. In line with our expectations about the importance of children for couples’ time synchronization, we estimate separate versions of equation for couples with and without children.

Similar to the above studies, we also control for (daily) hours of work in all specifications in order to separate the direct effects on synchronous time from indirect effects that may operate through the duration of work (there is more chance of schedules overlapping with longer working hours). Thus, by fixing the number of hours of work the effect of flexibility on synchronous working time reported here may be interpreted as a lower bound of the efforts made by couples to meet work-life balance issues, as the number of hours worked are decided on the basis of family needs.^9^

## Main results

### Flexitime vs. other forms of flexibility

Table 3 reports the baseline estimates, first for all couples and then breaking them down according to presence and age of youngest child. Results from a full specification shows that, although the woman’s flexitime coefficient is significant, while the man’s flexitime coefficient is close to zero, the woman’s coefficient is not significantly different from the man’s. We thus present specifications for a couple’s dummy taking value 1 if either partner has flexitime, as our data do not allow differentiating gender effects any further.^10^

**Table 3:** The impact of flexible work on couples’ synchronous working time

	All	No children	Youngest child under 5 years	Youngest child over 5 years
	(1)	(2)	(3)	(4)
Has flexitime (m or f)	0.527**	0.042	0.897**	0.874**
	(0.219)	(0.308)	(0.442)	(0.343)
Annualized hours (m or f)	–0.156	–0.037	–1.548**	–0.034
	(0.281)	(0.364)	(0.736)	(0.577)
Control over week hourrs (m or f)	0.000	0.318	–0.680	–0.425
	(0.252)	(0.345)	(0.651)	(0.488)
Youngest child <5 years	–1.279**			
	(0.297)			
Youngest child 5–11 years	–0.880**			
	(0.300)			
Youngest child 12–15 years	–0.490			
	(0.336)			
Daily work duration (f)	0.566**	0.564**	0.467**	0.569**
	(0.044)	(0.069)	(0.096)	(0.080)
Age/10 (f)	0.702	–0.513	2.986	1.489
	(1.221)	(1.447)	(3.713)	(4.006)
(Age/10)^2^ (f)	–0.080	0.059	–0.324	–0.180
	(0.153)	(0.179)	(0.538)	(0.472)
Degree (f)	1.311**	0.714	2.206*	1.115
	(0.494)	(0.687)	(1.251)	(0.823)
Further education (f)	0.380	0.521	–0.510	0.377
	(0.438)	(0.601)	(1.237)	(0.815)
A level (f)	0.921**	0.870	1.448	0.513
	(0.453)	(0.615)	(1.375)	(0.789)
O level or equiv (f)	0.833*	0.580	0.949	0.863
	(0.432)	(0.596)	(1.244)	(0.778)
Other qual (f)	0.829	1.099	–0.906	0.414
	(0.531)	(0.726)	(1.706)	(0.877)
Log hourly wage (f)	0.165	0.603**	0.313	–0.034
	(0.182)	(0.299)	(0.468)	(0.269)
Daily work duration (m)	0.326**	0.344**	0.306**	0.263**
	(0.053)	(0.081)	(0.092)	(0.086)
Age/10 (m)	–0.935	–0.677	–2.493	0.790
	(1.138)	(1.421)	(3.310)	(3.068)
(Age/10)^2^ (m)	0.098	0.072	0.325	–0.118
	(0.134)	(0.164)	(0.426)	(0.354)
Degree (m)	0.773**	0.480	0.904	1.289*
	(0.391)	(0.545)	(1.070)	(0.704)
Further education (m)	0.596*	0.446	1.575	0.536
	(0.324)	(0.415)	(1.027)	(0.673)
A level (m)	0.517	0.267	1.528	0.465
	(0.392)	(0.493)	(1.315)	(0.780)
O level or equiv (m)	–0.113	–0.324	0.233	0.158
	(0.357)	(0.503)	(1.107)	(0.698)
Other qual (m)	0.403	1.015	0.454	–0.394
	(0.619)	(1.014)	(1.280)	(0.797)
Log hourly wage (m)	0.420**	0.392	0.209	0.315
	(0.168)	(0.239)	(0.399)	(0.370)
*N* (couples)	1523	772	256	495

OLS estimates at couple level, weighted for survey design and non-response. Standard errors in parentheses. O-level (or equivalent) and A-level are the school leaving certificates obtained at 16 and 18 years respectively. Models include dummy variables for region

* Significant at 10% level

** Significant at 5% level

Confirming our hypothesis that flexible work facilitates hours coordination, column 1 in Table 3 shows that among all couples (column 1), those with flexitime synchronize their work schedules by about half an hour per day more than couples without flexitime. But in contrast to flexitime, neither annualized hours nor general control over working hours is associated with more synchronous working time.^11^ A possible reason is that annualized hours may involve changes in the length of the working week at certain periods of the year rather than adjustment on a daily basis, and so may not show up in daily synchronous time. The data do not enable us to evaluate time coordination over the week (or longer periods) but only the day.

Similarly, it could be that working hours control captures a broader notion of flexibility than flexitime, including both the timing and amount of work (the survey question refers only to “the hours you work”). The data show that only 27% of those reporting working hours control also report flexitime, while almost 90% of those with flexitime also report working hours control. Thus control over working hours may involve flexibility over a different timescale to flexitime and any impacts may be reflected less strongly in our measure of daily synchronous time.

Alternatively, our results can be interpreted as an indication that measures of annualized hours and general control over working hours may not necessarily give workers more control over their working time; to the contrary, they may be associated with more managerial control (Heyes 1997) and firm-orientated flexibility (Messenger 2011; Kerkhofs et al. 2008), rather than worker-oriented flexibility. Indeed, among couples with children under 5 years (column 3), annualized hours seem to be associated with less synchronous time. Flexible work arrangements may indeed be a double-edged sword. Whilst rigid work schedules effectively ring-fence leisure and family time, flexible work patterns carry the danger that all time is potentially work time. Flexible work has indeed been found to be associated with longer total work hours (Golden 2001) and with work intensification (Kelliher and Anderson 2010), possibly because employees repay increased flexibility with greater effort.

The sign and size of the coefficients on the other controls are in line with previous research. Higher educated and higher earning couples tend to synchronize more. For example, among the all-couple sample the possession of a degree by either spouse is associated with more synchronous time (1.3 h per day when the graduate is a woman and 0.8 h when the man holds a degree). Higher men’s hourly wages are also associated with more synchronous time, although there is no significant effect of higher women’s wages (except in couples without children). As noted by Hamermesh (2002), the wage coefficient may combine two effects: a positive income effect (since, holding hours constant, hourly wages reflect full earnings) and a negative price effect if wages are lower for work schedules that allow synchronization. Our results indicate that the income effect is at least as large as any price effect: overall, higher income couples demand more synchronous time. The findings for wage effects in previous studies are mixed, possibly because of the two opposing effects, but there is no evidence from these other studies that higher earners synchronize their work less.^12^ An alternative explanation is that couples with lower incomes need to stagger their work hours so that one of them is doing childcare to avoid paying someone else. As expected longer work durations lead to more work synchronization, and women are more likely to increase their work hours during their partner’s working time than are men (for couples as a whole, the coefficient on women’s work hours is 0.57 compared with 0.33 for men).

### The role of children

In line with our expectations, and consistent with most previous studies (Hamermesh 2000; van Klaveren and van den Brink 2007; Jenkins and Osberg 2005; Scheffel 2010),^13^ we find a strong negative association between having children and couples’ synchronous work time. We see this relationship especially for young children, in particular those under 5, whose parents have 1.3 h less synchronous time per day compared to couples without children (column 1). This result can be easily rationalized in our model. To the extent that a couple’s coordination is a means to achieving the goal of producing higher quality children, having children reduces synchronous time via the *X* factors in Eq. (2). These factors include rigidities in provision of market childcare may lead couples to make sure that one parent is always present with the child (and they may not be able to afford paid childcare) and so they stagger their work start and finish times (Fermanian and Lagarde 1999). Similarly, the timing-sensitive nature of children’s routines may also affect parents’ time scheduling at home and at work, for instance a child’s nap allows time to relax but not necessarily at the best time for both parents (Barnet-Verzat et al. 2011).

We next look compare the effect of flexible work for couples with and without children. Columns 3 and 4 in Table 3 shows that the positive relationship between flexitime and synchronous time is being driven by couples with children, for whom flexitime allows nearly 1 h per day more of synchronicity. In contrast, column 2 shows that there is however no effect of flexitime among those without children.^14^

Again, we can rationalize these results in our model. Having children reduces the choice set of schedules that couples have available, such that for example *H* = {*h*_1_, *h*_3_} rather than {*h*_1_, *h*_2_, *h*_3_}, in turn reducing the ability to synchronize a couple’s working time. The reduction in the choice set of schedules for couples with children may be a result of less geographical mobility relative to couples without children. For example, parents may be reluctant to move jobs because their children would have to move schools. Our data indicate this could be a plausible mechanism: couples with children of school age are much less likely to move house (7% moved since the previous wave) than those without children (12%) (as also found by Rabe 2011). In addition, the timing-sensitive nature of childcare needs may coincide with a thinner distribution of work schedules at those times which are most important for childcare, for example fewer jobs finish at 3 pm than at 4:30 pm or 5 pm (and 4:30 pm may be too late to collect children from school).

With a smaller choice set of schedules, couples are likely further away from their optimal degree of synchronization, and thus flexible work potentially has a larger role to play in adjusting their synchronous time. In contrast, our results suggest that the work schedules of couples without children are sufficient for them to coordinate freely (up to their desired level of synchronziation), and thus flexible work makes no difference to their synchronous time.

### Understanding the mechanisms

In light of the importance of having a job with flexitime for couples with children, we now investigate in more depth what may lie behind the strong positive association of flexitime and synchronization for couples with dependent children. A possible mechanism is selection. Individuals with a greater preference for synchronizing with their partners may select into industries with more flexible jobs (such as the public sector). They may also choose occupations where flexitime is more common, and which may also involve more standardized work schedules (e.g., daytime work rather than night shifts) that lead automatically to greater synchronization. By successively introducing industry and occupation controls into the regressions, we can assess the importance of these channels.

To evaluate the extent of selection we estimate Eq. (3) controlling first for industry and sector (public/private), and then adding in occupation (all controls are included for both partners). Although these additional controls are clearly endogenous, the goal of this exercise is to gauge how much of the flexitime effect they explain. Table 4 shows that the effect of flexitime on synchronization previously shown for couples with dependent children (Table 3) is robust to controlling for industry and sector (columns 1 and 3). Thus the flexibility effect on couples’ synchronization are not driven by couples selecting into industries or sectors that offer more flexitime. Neither is the effect of flexitime on couples’ synchronization driven by occupational selection for couples with the youngest child over 5 years. Column 4 shows that flexitime allows couples with older children to synchronize 0.7 h more per day than couples with older children but without flexitime (column 4). By contrast there is some evidence of occupational sorting for couples with children under 5. The flexitime coefficient falls to about half its previous value and is not precisely estimated (although a caveat here is that the degrees of freedom are reduced quite dramatically as the sample size is only 256 and there are 48 occupation/industry/sector controls).^15^

**Table 4:** The impact of flexible work on couples’ synchronous working time, by age of children, controlling for industry, sector and occupation

	Youngest child under 5 years		Youngest child over 5 years	
	(1)	(2)	(3)	(4)
Has flexitime (m or f)	0.973**	0.467	0.781*	0.722*
	(0.478)	(0.516)	(0.401)	(0.388)
Annualized hours (m or f)	–2.070**	–1.740**	–0.186	–0.084
	(0.722)	(0.628)	(0.528)	(0.515)
Control over work hours (m or f)	–0.533	–0.671	–0.363	–0.210
	(0.579)	(0.576)	(0.506)	(0.498)
Industry and sector controls	Yes	Yes	Yes	Yes
Occupation controls	No	Yes	No	Yes
*N* (couples)	256	495		

OLS estimates at couple level, weighted for survey design and non-response. Standard errors in parentheses. Models include (for each spouse) daily work duration, age and age squared, highest educational qualification, log hourly wage, and region. Industry/sector controls consist of 15 dummy variables based on SIC92 divisions and a dummy variable for public vs private sector. Occupation controls are 8 occupation dummy variables based on SOC 1990 major groups. Industry/sector and occupation enters separately for each spouse. The number of couples with flexitime is 48 (in columns 1 and 2) and 78 (columns 3 and 4)

* Significant at 10% level

** Significant at 5% level

### Addressing the potential endogeneity of flexitime

Even within industries and occupations, partners who want to spend time together may seek out jobs with more flexitime, that allow, for example, working at home or bringing work home. To assess this channel we use an instrumental variables (IV) strategy using aggregate geographical variation in the availability of flexible work as a predictor of a couples’ flexible work.^16^ In particular we use local government administrative areas, i.e., Local Authority District (LAD) areas, for which identifiers can be obtained for the BHPS, and calculate the proportion of workers with flexible work in a given local area unit (excluding the sample year from the calculation). There are a total of 406 such areas in the UK.^17^ The resulting aggregate variable has a mean 0.14 and standard deviation of 0.13, thus there is a reasonable amount of variation across areas.

For this instrument to be valid, it should be unrelated to unobserved determinants of synchronous time. This seems a tenable assumption unless people move to areas with more flexible jobs in order to have more synchronous time. Given that spouses live together, such a move would have to involve both partners relocating simultaneously.

Table 5 shows OLS estimates of the new specification (columns 1 and 3), breaking down the sample by age of youngest child. As we have a single instrument, the specification only includes flexitime (i.e., a single endogenous variable), excluding the other flexible work measures that were included in the benchmark specification (Table 3). Results are similar to the coefficients in Columns 3 and 4 in Table 3 as expected, given that the other two flexible work measures were not significant in that specification.

**Table 5:** The impact of flexitime on synchronous working time, couples with dependent children (endogenous flexitime)

	Youngest child under 5 years		Youngest child over 5 years	
	OLS	IV	OLS	IV
	(1)	(2)	(3)	(4)
Has flexitime (m or f)	0.774*	1.866*	0.752**	–1.648
	(0.448)	(1.085)	(0.357)	(1.373)
*F*-test of excluded instrument		28.9		26.0
Hausman test of equal flexitime coefficients χ_2_ (1)		0.02		1.64
*N* (couples)	256	256	495	495

OLS estimates at couple level, weighted for survey design and non-response. Standard errors in parentheses. Models include (for each spouse) daily work duration, age and age squared, highest educational qualification, log hourly wage, and region. Instrument is proportion of flexitime in local authority district. Hausman test carried out on unweighted estimates. Standard errors in parentheses

* Significant at 10% level

** Significant at 5% level

The two-stage least squares estimates are shown in columns 2 and 4. Both first-stage coefficients are highly significant, with *F* tests of instrument exclusion of 28.9 and 26.0 (which are both well above the critical values for weak identification derived by Stock and Yogo 2005). The IV coefficients in Columns 2 and 4 show that having flexitime increases work synchronization by 1.9 h for couples with a child younger than five, but it is not significant for couples with the youngest child older than five. However, Hausman tests do not find differences between either of these IV coefficients and the corresponding OLS estimates in Columns 1 and 3 in Table 5. Overall then, our best causal estimate of the flexitime effect suggests that the OLS results are not just reflecting either selection into jobs with more flexitime by couples with a higher taste for work synchronization, or reverse causality.

## Conclusion

In recent years there has been increasing interest in flexible work as a means to help households reconcile work and family commitments, have quality time together as a family, and generally improve their work–life balance. This paper focuses on how work time arrangements relate directly to the synchronization of work schedules between the partners in a couple.

We exploit innovative information regarding the times that partners start and finish work in Britain, together with measures of three specific types of flexible work arrangement. We show that, after controlling for a wide range of co-founders, flexitime is associated with a half to 1 h increase in daily synchronous time. In contrast to flexitime, neither a broader measure of work hours control nor annualized hours are strongly associated with work schedule coordination.

We also find that the positive effect of flexitime on couple’s synchronization of work schedules is driven by couples with young children. This finding suggests that couples with children may face a variety of additional constraints in the labor market with respect to couples without children; and as a result the ability of parents to choose working schedules may be lower than the ability of non-parents to achieve the desired amount of work synchronization. In the face of scheduling constraints faced by families with children, flexitime can thus be a valuable mechanism for couples with young and older children to fine-tune schedules to their needs and so regain some synchronous time.

The implications of our findings for further research and practice are of great interest given the expansion of flexibility policies. The ‘right to request’ flexible work has gradually been extended over the past decade in the UK and as of June 2014 is available to all employees.^18^ Nonetheless recent figures indicate that only 48% of employees perceive that flexitime is available in their workplace (Tipping et al. 2012). Hence our findings suggest that an extension of flexitime would be a promising route toward more synchronous family time, especially among parents.

There are some limitations to our research. First, our results are limited to the effect on a couple’s synchronous time from the three forms of flexible working captured by the BHPS. Other dimensions of flexibility, such as the ability to work from home, are not collected by the BHPS. In our analysis we control for a wide set of co-founders, such as industry and sector. To the extent that these other work flexibility dimensions are industry and sector-specific, our results should hold. It would however be interesting to see whether other forms of flexible working affect the ability of couples to synchronize their daily activities.

Second, our measure of couple’s synchronization of work schedules is a measure of overall joint non-working time and not time spent in specific home activities or in commuting. All else equal, the potential time that the couple can be together outside working hours is greater the more they synchronize their work schedules, but even so they may choose to spend their time separately. Testing whether flexitime affects the breakdown of activities, as opposed to overall non-working time, will allow us to further disentangle the mechanisms by which children of different ages affect the ability of couples to coordinate and the mediating role of flexible time policies within the firm. To that end, diary data, which follows individuals throughout a 24 h period and has information on work schedules and time together as a couple, will need to be collected together with information on whether the respondent has job flexibility. A better understanding of these questions provides an interesting avenue for further research.

## Appendix 1

1.1 Construction of synchronous time measures

Table 6 gives the wording of the work timing questions used in the analysis, taken from wave 13 in the BHPS. The questions routing is as follows. First, respondents were asked about which type of schedule they worked. Second, depending on the type of work schedule, they were asked about the hours that they worked each day. Those working the same hours each day (whom we term *regular workers*) were asked for the times they usually started and finished work, for up to 3 separate daily periods (for example, morning, afternoon and evening). Respondents working rotating shifts or with no fixed pattern (*irregular workers*) were asked to report start and finish times on each day of the week just ended (one period per day), including the weekend. Among the sample of dual earner couples, 58% both work regular hours, 34% contain one regular and one irregular worker and 8% both work irregular hours.

**Table 6:** Questions about work timing in the BHPS Wave 13

Variable Name	Question	Answer
MJBWKPT	“Thinking about your (main) job, do you usually work the same hours each day, work rotating shifts or is there no fixed pattern?”	The same hours each day
		Rotating shifts
		No fixed pattern
MJBST*H, MJBST*M, MJBEN*H, MJBEN*M; * = 1–3 (for respondents working the same hours each a day)	“Would you please tell me at what times you usually start and finish work. If you have multiple spells of work in a day, for example, some hours in the morning and some in the evening, please tell me the start and finish time of each work period.”(Include lunch breaks within one work period)	Start time (hours, minutes)End time (hours, minutes) For up to three periods
Variables MLWST*H, MLWST*M, MLWEN*H, MLWEN*M, MLWDNW*; * = 1–7 (for respondents working rotating shifts or no fixed pattern)	“Thinking about the times you worked in the week ending last Sunday, can you tell me your start and finish times for each day starting on the previous Monday.”	Start time (hours, minutes)
		End time (hours, minutes)
		Didn’t work that day
		Don’t know
		For each day Monday–Sunday

Source: Wave 13 BHPS

The vast majority of regular workers, over 92%, reported just one daily period (for example, 8am–5 pm), 8% reported two periods and 1% reported three periods. One issue is that about half of those reporting multiple periods gave periods which overlapped (for example, 8am to 3 pm and 9am to 4 pm). Although these individuals reported (in the previous question) that they worked the same hours each day, their answers suggest that their shift times may in fact change. Owing to a lack of further information about how these shifts change day by day, we treat these responses as invalid and omit any couple containing such individuals (6% of couples).

Calculating our measure of synchronous time is a straightforward process for couples in which both partners work regular hours each day. For example, if one spouse works from 9am to 6 pm and the other from 7am to 3 pm, their synchronous work time is from 9am to 3 pm, or 6 h. For couples with two irregular workers, we match their schedules for each joint working day from Monday to Friday in turn, and then calculate the mean synchronous time over these days (assuming that the week reported represents a typical week). The matching process is more complicated for couples with one regular worker and one irregular worker. We assume, in the absence of more information, that regular workers work Monday to Friday.^19^ We then match the regular shift to the irregular work times given by the spouse for each day in turn from Monday to Friday. Our final measure of synchronous time is the mean synchronous time calculated over the days that both spouses work. For example, suppose the spouse on regular hours works from 9am to 6pm, and the other spouse works on two days: 7am to 3pm on Monday, and 1pm to 9pm on Wednesday. Synchronous time is 6 h for Monday (9am to 3pm)) and 5 h forWednesday (1pm to 6pm), so synchronous time for a typical joint working day is 5.5 h.

**Table 7:** Questions about flexible work in the BHPS Wave 13

Variable Name	Question	Answer
MJBWKHRA, MJBWKHRB	“Some people have special working hours arrangements that vary daily or weekly. In your (main) job is your agreed working arrangement any of those listed on the card.”	Flexitime (flexible working hours); Annualized hours contract; Term time working; Job sharing; 9-day fortnight; Four-and-a-half day week; 0 h contract; None of these
MJBPATW	“Thinking about the hours you work in your main job, which of the statements on this card best describes your situation?”	Your employer decides the hours you work; Your employer decides the hours you work but there is some flexibility; You and your employer jointly decide the hours you work; You decide the hours you work

Source: Wave 13 BHPS

**Table 8:** Means (standard deviations) of variables

(a) Male and female spouses		
Variable	Men	Women
Daily work duration (hours)	9.04	7.13
	(1.94)	(2.54)
Works flexitime	0.15	0.19
Annualized hours	0.09	0.07
Control over working hrs	0.54	0.58
Age (years)	41.56	39.66
	(9.93)	(9.75)
Degree	0.17	0.19
Further education	0.40	0.36
A level	0.13	0.11
O level or equiv	0.15	0.18
Other qualification	0.06	0.09
No qualification	0.08	0.07
Qualification missing	0.02	0.01
Hourly wage (£)	13.91	10.44
	(8.04)	(7.73)
Manager	0.22	0.11
Professional	0.10	0.10
Technician	0.11	0.14
Clerical	0.08	0.27
Craft	0.19	0.01
Personal	0.06	0.15
Sales	0.04	0.09
Operative	0.13	0.03
Routine	0.05	0.07
Agriculture	0.00	0.00
Mining	0.01	0.00
Manufacturing	0.27	0.08
Utilities	0.02	0.01
Construction	0.07	0.01
Retail	0.12	0.14
Hotels	0.02	0.02
Communications	0.10	0.04
Finance	0.04	0.06
Property	0.11	0.10
Public administration	0.09	0.09
Education	0.05	0.18
Social work and health	0.04	0.22
Other industries	0.04	0.04
Private households	0.00	0.00
Extra-territorial organizations	0.01	0.01
Public sector	0.19	0.42

(b) Couples	
Variable	
Daily synchronous working time (hours)	5.67
	(3.40)
Children under 16 in household	0.50
North-East	0.05
North-West	0.12
Yorkshire	0.09
East Midlands	0.09
West Midlands	0.08
East	0.09
South East	0.15
South West	0.09
Wales	0.04
Scotland	0.08
Northern Ireland	0.02

All estimates are weighted for survey design and non-response. Unweighted base is 1523 couples

## Footnotes

1 Jenkins and Osberg (2005, p. 132) found that couples with one or two children were more likely to have synchronous work times than similar pseudo-couples (with one or two children), while couples with three or four children were less likely. Couples with one or two children represented 87% of couples with children in their sample.

2 We define synchronous (work) time as the length of time during which both partners are at work simultaneously (per day). An alternative measure would be “synchronous home time”, the amount of time they can potentially spend together outside of work. The two are linked by the identity: synchronous home time = 24 h – work duration(m) – work duration(f) + synchronous work time. As we control for work duration in our multivariate analysis, it effectively makes no difference which measure we use, i.e., conditional on work duration the determinants of synchronous home and synchronous work time are identical.

3 All analyses are weighted to account for inclusion of the extension samples and for non-response.

4 Part-time work is defined as 30 h per week or less.

5 The work overlap variable contains 243 discrete values, the most common being zero hours (13%), 8 h (11%), and 7.5 h (6%). None of the other values exceeds a frequency of 5%.

6
https://www.gov.uk/flexible-working/types-of-flexible-working

7 We also estimate specifications controlling for whether the couple is married as opposed to cohabiting. The results do not change.

8 The models reported include all three flexible work measures. We obtain very similar results (available on request from the authors) if we estimate equations that include each flexible work measure separately.

9 Separate estimates for dual FT and FT-PT couples are not statistically significantly different from each other.

10 The symmetry between man’s and woman’s flexitime effects contrast to what has been found in the retirement literature, where a woman’s retirement increases joint leisure but not the man’s retirement (Stancanelli and van Soest 2016). These contrasting results may be explained by the different time constraints faced by retired couples versus two-earner couples.

11 There is some evidence from the extended specification that men’s, but not women’s, control over working time is associated with more synchronous time. But as the two effects do not differ significantly, we prefer the couple-level measure on statistical efficiency grounds and conclude there is not robust evidence of a relationship between generalised hours control and synchronous time.

12 Hamermesh (2002) found robust evidence that higher earners synchronized more, but Hallberg (2003, p.199) reported that income did not affect synchronous time, while Jenkins and Osberg (2005) found that only the woman’s (and not the man’s) wage rate affected synchronous time (they controlled for the man’s education but not the woman’s and as noted used a binary indicator of synchronisation).

13 In contrast, Hallberg (2003) finds that children increase synchronous housework but reduce synchronous leisure, with no net effect on overall synchronous time.

14 We also estimated an equation for all couples with children and tested whether the flexitime coefficient differed from the equation without children (Table 3, column 2). The difference was significant (*p* = 0.016). Full estimates available from the authors on request.

15 We also included interactions of flexible work with dummies for blue and white collar couples, but did not find differential effects by occupational status.

16 We also considered as alternative instruments job characteristics from before the couple formed, based on the idea that these characteristics (i) predict access to flexible work but (ii) are not be related to the couple’s desire for time together. Unfortunately, the second condition will not be satisfied in the likely event that a person’s choice of job is related to their general taste for leisure. Moreover, using the BHPS marital/fertility histories, we can only trace detailed pre-partnership characteristics for 20% of sample or 300 couples (for the others, the start of partnership is censored in the marital history data or the partnership began before the panel started). We also considered parental background (occupation when the respondent was 14) as a predictor of access to flexible work. While this may be theoretically valid, in practice parental background does not predict flexible work at all.

17 The breakdown of areas is 326 LADs in England, 22 Unitary Authorities in Wales, 32 Council Areas in Scotland and 26 District Council Areas in Northern Ireland.

18
https://www.gov.uk/flexible-working/overview. An employer can refuse a flexible work application if they have a good business reason for doing so.
